# Stroke-Related Mortality at Different Altitudes: A 17-Year Nationwide Population-Based Analysis From Ecuador

**DOI:** 10.3389/fphys.2021.733928

**Published:** 2021-09-30

**Authors:** Esteban Ortiz-Prado, Patricio S. Espinosa, Alfredo Borrero, Simone P. Cordovez, Jorge E. Vasconez, Alejandra Barreto-Grimales, Marco Coral-Almeida, Aquiles R. Henriquez-Trujillo, Katherine Simbaña-Rivera, Lenin Gomez-Barreno, Gines Viscor, Paul Roderick

**Affiliations:** ^1^One Health Research Group, Faculty of Medicine, Universidad de Las Américas, Quito, Ecuador; ^2^Departamento de Biología Celular, Fisiología e Inmunología, Universitat de Barcelona, Barcelona, Spain; ^3^Neurology, Marcus Neuroscience Institute, Boca Raton Regional Hospital, Boca Raton, FL, United States; ^4^Faculty of Medicine, School of Primary Care, Population Sciences and Medical Education, University of Southampton, Southampton, United Kingdom

**Keywords:** stroke, high altitude, mortality, angiogenesis, adaptation, Ecuador

## Abstract

**Introduction:** Worldwide, more than 5.7% of the population reside above 1,500 m of elevation. It has been hypothesized that acute short-term hypoxia exposure could increase the risk of developing a stroke. Studies assessing the effect of altitude on stroke have provided conflicting results, some analyses suggest that long-term chronic exposure could be associated with reduced mortality and lower stroke incidence rates.

**Methods:** An ecological analysis of all stroke hospital admissions, mortality rates, and disability-adjusted life years in Ecuador was performed from 2001 to 2017. The cases and population at risk were categorized in low (<1,500 m), moderate (1,500–2,500 m), high (2,500–3,500 m), and very high altitude (3,500–5,500 m) according to the place of residence. The derived crude and direct standardized age-sex adjusted mortality and hospital admission rates were calculated.

**Results:** A total of 38,201 deaths and 75,893 stroke-related hospital admissions were reported. High altitude populations (HAP) had lower stroke mortality in men [OR: 0.91 (0.88–0.95)] and women [OR: 0.83 (0.79–0.86)]. In addition, HAP had a significant lower risk of getting admitted to the hospital when compared with the low altitude group in men [OR: 0.55 (CI 95% 0.54–0.56)] and women [OR: 0.65 (CI 95% 0.64–0.66)].

**Conclusion:** This is the first epidemiological study that aims to elucidate the association between stroke and altitude using four different elevation ranges. Our findings suggest that living at higher elevations offers a reduction or the risk of dying due to stroke as well as a reduction in the probability of being admitted to the hospital. Nevertheless, this protective factor has a stronger effect between 2,000 and 3,500 m.

## Introduction

Cerebrovascular disease or stroke is the second leading cause of death worldwide; affecting more than 16 million people each year (Hankey, 2017). Around one in six men and one in five women will have a stroke in their lifetime, In 2016, the global lifetime risk of stroke from the age of 25 years onward was approximately 25% among both men and women (Global Burden of Disease group, 2018). Stroke is the third leading cause of disability worldwide and affects people of all ages, though the causes of stroke at a younger age are very different from those at older ages (Fullerton et al., 2003; Seshadri and Wolf, 2007; Johnson et al., 2016; deVeber et al., 2017). The risk of developing stroke increases with high blood pressure, atrial fibrillation, cigarette smoking, hyperlipidemia, and diabetes mellitus (Hankey, 2017). Other modifiable factors are obesity, chronic kidney disease, excessive alcohol use, cocaine consumption, sedentarism, psychological stress and depression (Everson et al., 1998; Cheng et al., 2016; Guzik and Bushnell, 2017). The list of non-traditional factors linked to stroke includes some environmental conditions such as high altitude exposure. Hypobaric hypoxia due to living in mountainous regions may play a role in stroke incidence and mortality; nonetheless, this environmental factor has been poorly investigated (Jha et al., 2002; Niaz and Nayyar, 2003; Pilz et al., 2008; Szawarski et al., 2012; Gürdal et al., 2018).

Worldwide, at least 5.7% of the population live above 1,500 m, with millions of people chronically exposed to high altitude (Tremblay and Ainslie, 2021). There are regions of the world with millions of people living above 2,500 m, including the South American Andes, the Indochinese Himalayas and the Ethiopian Plateaus (Tremblay and Ainslie, 2021). The association between high altitude exposure and stroke is still unknown and the very few investigations available are still inconclusive (Jha et al., 2002; Niaz and Nayyar, 2003; Ezzati et al., 2012; Burtscher, 2014; Mallet et al., 2021). It has been difficult to define at which elevation the effects of high altitude become more severe and where the threshold is located in terms of mild or severe hypoxia (West et al., 2007). The International Society of Mountain Medicine defines low altitude everything located below 1,500 m, moderate or intermediate altitude between 1,500 and 2,500 m, high altitude from 2,500 to 3,500 m, the very high altitude from 3,500 to 5,800 m, more than 5,800 extreme high altitude and above the 8,000 m is considered the death zone (Imray et al., 2011).

Anecdotal evidence suggest that acute exposure to high altitude (>2,500 m) might increase the risk of thrombosis secondary to short-term hypoxia, which has been associated with the development of ischemic stroke (Kotwal et al., 2007; Gupta and Ashraf, 2012; Zangari et al., 2013). Most of these studies found a significant association between living in high altitude and having a higher risk of stroke, especially among younger populations (<45 years of age) (Jaillard et al., 1995; Jha et al., 2002; Niaz and Nayyar, 2003; Faeh et al., 2009). Contrasting results were published by Faeh et al. (2009) who found a decreased risk of cardiovascular diseases (CVD) and stroke-related mortality among those living in high altitude locations in Switzerland (Faeh et al., 2009). This study reported a 12% decreased risk of cardiovascular diseases and stroke-related mortality per 1,000 m of elevation according to mortality data from the year 1990 to 2000. The dataset included sociodemographic information, place of birth and place of residence as well as the median elevation of each city, ranging from 259 to 1,960 m (Faeh et al., 2009).

Faeh et al. (2016) conducted a well-controlled analysis in Switzerland during 2016 through investigating whether changes in temperature, terrain characteristics, and built environment muddled the relationship between living at a moderately higher altitude and having a decreased risk of IHD. After accounting for all other environmental parameters, they revealed that the inverse altitude-IHD relationship maintained, and probably physical environment factors appear to have an independent effect on cardiovascular health (Faeh et al., 2016).

Although this offers a new perspective of the potential protective effect of living at higher altitudes, the elevation range did not surpass 2,000 m, making it difficult to extrapolate the results to other mountainous regions of the world. To further explore the relationship between high altitude and stroke, we conducted a nationwide ecological study in Ecuador with data from 2001 to 2017, including more than 100,000 stroke patients living at different elevations, ranging from 0 m at sea level to 4,300 m within the Ecuadorian highlands.

## Materials and Methods

### Study Design

This is an ecological analysis of the geographical distribution of stroke using hospital admissions as a proxy for incidence and stroke mortality in Ecuador from 2001 to 2017. The analysis included all the stroke cases and fatalities reported in every city (cantons) of Ecuador as the unit of analysis with a yearly resolution. Stroke cases included all the hospital admissions and deaths according to the patient’s place of residence reported to the National Institute of Census and Statistics (INEC).

### Sample and Setting

**FIGURE 1:**
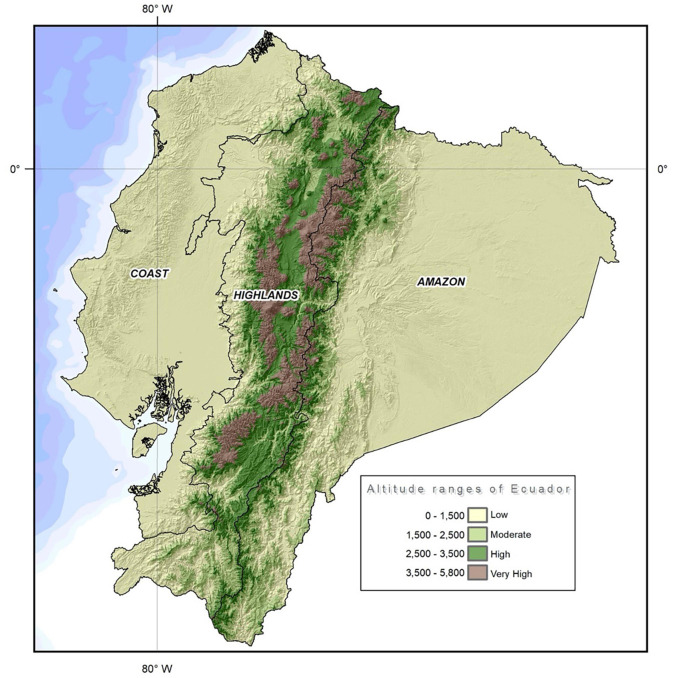
Topographic map of Ecuador using elevation contour lines to show the shape of the country’s surface. Map created by the Authors.

A country-wide comparison of the total number of strokes from the 24 provinces and the 221 cantons in Ecuador was performed from 2001 to 2017. Ecuador with an area of more than 283,000 km^2^ is the smallest country in the Andean mountainous region in South America. The country is divided into four geographical regions, the coast, the highlands, the Amazon region, and the Galapagos Islands. The political division encloses 24 provinces, 10 from the highlands, seven from the coast, six from the Amazon region, and one from the insular region of Galapagos. Every province has several political divisions called cantons and they are comparable to cities elsewhere. The country has 141 cantons at low altitude, 28 at moderate altitude, 41 at high altitude, and 11 at very high altitude (Figure 1).

### Population

**FIGURE 2:**
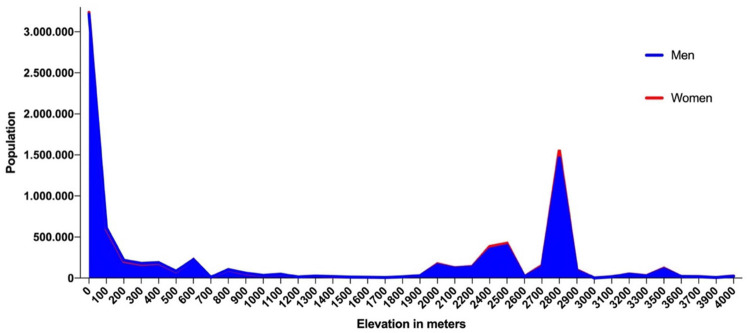
Population by elevation in Ecuador by sex.

According to the 2017 National Institute of Census and Statistics (INEC) data projections, Ecuador has a population of 17,082,730, 51% women and 49% men. In terms of ethnicity, most of people are Mestizo (79.3%), followed by Afro-Ecuadorians (7.2%), indigenous (7.1%), white or Caucasian descendants (6.1%) and other groups (0.4%) (INEC, 2010). By elevation, Ecuador has 60% of its population residing at low altitude (<1,500 m), 10% at moderate altitude (1,500–2,500 m), 27% at high altitude (2,500–3,500 m) and 3% at very high altitude (3,500–5,500 m) (Figure 2).

### Exposure

The association between altitude exposure and stroke incidence and mortality was analyzed. The classification of low altitude <2,500 m and high altitude >2,500 m was used as a cut-off point for elevation exposure, while the classification offered by the International Society of Mountain Medicine (low altitude (<1,500 m), moderate altitude (1,500–2,500 m), high altitude (2,500–3,500 m) and very high altitude (3,500–5,500 m) was used to assess prevalence odds ratios by different elevations (Imray et al., 2011).

### Outcome

Stroke age-sex and altitude adjusted incidence and mortality rates were calculated using the total number of stroke hospital admissions and all the stroke-related deaths in Ecuador.

### Data Source

Data were retrieved from the National Institute of Census and Statistics (INEC) using the general hospital admission and the mortality databases from the last 17 years of available data on discharges and death certificates according to the patient’s place of residence within the public and private health system in Ecuador. The databases included the latest International Classification of Diseases 10th Revision (ICD-10) coding system and the information concerning stroke was retrieved from the INEC public domain at https://aplicaciones3.ecuadorencifras.gob.ec/sbi-war/.

Data concerning hospitals beds, doctors, and hospital per capita were obtained from the health-related activities database from 2018, available from the following public domain at https://www.ecuadorencifras.gob.ec/actividades-y-recursos-de-salud/.

### Inclusion Criteria

Using the International Classification of Diseases 10th Revision (ICD-10) the following subtypes of stroke cases and deaths were included: I60 subarachnoid hemorrhage (SAH), I61 intracerebral hemorrhage (ICH), I63 ischemic stroke, I64 Stroke not specified, and the combination of all of them in a new variable called “all strokes”.

### Exclusion Criteria

Patients without an ICD-10 diagnosis of major stroke were not included as Kokotailo and Hill defined “major” stroke types to those described as I60, I61, I63, and I64 (Kokotailo and Hill, 2005). The following ICD-10 codes including transient ischemic attack (TIA) were excluded: I65 Occlusion and stenosis of precerebral arteries, not resulting in cerebral infarction, I66 Occlusion, and stenosis of cerebral arteries, not resulting in cerebral infarction, I67 Other cerebrovascular diseases, I68 Cerebrovascular disorders in diseases classified elsewhere and I69 Sequelae of cerebrovascular disease.

### Bias

To reduce the possibility of incurring in some degree of selection bias and due to the nature of the data, two researchers (EOP and KSR) downloaded the dataset and ran the analyses independently. To ensure that the data pertained to persons residing at different altitudes, the variable “place of residence” was used instead of the variable “place of medical attention”.

### Statistical Analysis

Incidence and mortality crude and age-sex adjusted rates were calculated using the population at risk for every altitude location. The 2010 Ecuadorian census data were used as the standard population for the direct standardization (INEC, 2010). We have used the 2010 census data as the standard population since no real door-to-door data has been collected in subsequent years. Measurements of frequency (counts, absolute, and relative percentages), central tendency (mean and median), and dispersion (range and standard deviation), as well as absolute differences were performed for age, sex, and the canton’s elevation.

To reduce the impact of age-sex population distribution’s differences at different altitudes, a direct standardization method was applied to calculate expected incidence and mortality rates. The age-specific mortality rates (observed) for each age group in a given population was used to compute the age-specific expected mortality rates. By this method, we obtained the expected deaths for each age group of each elevation range to later add the number of expected deaths from all age groups and divide the total number of expected deaths by the standard population (Naing, 2000). A Poisson regression was used to find the altitude effect on incidence/mortality after adjusting for age and sex. For association, we obtained OR for the total number of expected cases by the population at risk in all the groups to obtain the likelihood of death due to stroke hospital admissions. Poisson regression models were used to quantify the association between sex, altitude, age, and the risk of stroke. Relative risks were obtained from the exponents of the coefficients of the corresponding models.

The analysis of the data employed SPSS statistics software for Macintosh (IBM Corp., 2014, version 24.0, Armonk, NY, United States) and the Poisson analysis was done in R version 3.6.2. Figures and graphs were performed in Prism 8 GraphPad Software version 8.2.0 (San Diego, CA, United States). The basic cartography maps were generated using QGIS Development Team 2.8 (Creative Commons Attribution-ShareAlike 3.0 license CC BY-SA).

### Ethical Consideration

This secondary data analysis of publicly available, anonymized data received ethical approval from the University of Southampton with the Faculty of Medicine Ethics Committee ERGO 51422.R3 number. None of the data used can be identified with any personal information as the dataset did not include names, addresses, e-mails, GPS locations, or telephone numbers. Since this secondary data is available on the government official websites, no individual codes or numbers were given, making it impossible to re-assess or reverse any data toward an individual.

## Results

From 2001 to 2017, a total of 38,201 deaths and 75,893 hospital admissions due to stroke (I60, I61, I62, and I64) were reported to INEC. The sex distribution for the deceased was 19,163 deaths for men and 19,038 for women. In terms of hospital admissions, men accounted for 52% (*n* = 39,569) and women 48% (*n* = 36,324) of hospital stroke admissions. Sex was not a risk factor for stroke when female is used as a reference [OR: 1.01 (0.99–1.028), *p*-value: 0.434].

We found that patients who reside at high altitude develop stroke at a later age than the low altitude dwellers (Table 1).

**TABLE 1:** Descriptive analysis of age median (in years) between deaths and hospital admission due to all-causes of stroke in Ecuador at different elevation ranges.

	Mortality				Hospital admission			
	Median	IQR	Median	IQR	Median	IQR	Median	IQR
				
	Men		Women		Men		Women	
<2,500 m	72	(72–74)	76	(76–77)	66	(66–67)	68	(68–69)
>2,500 m	76	(76–78)	79	(78–81)	69	(69–70)	71	(71–72)
Low altitude	71	(71–72)	75	(75–76)	66	(66–67)	68	(67–68)
Moderate altitude	77	(76–79)	78	(77–81)	68	(67–70)	73	(73–74)
High altitude	75	(74–79)	79	(78–81)	69	(69–70)	71	(71–72)
Very high altitude	78	(74–82)	77	(73–83)	67	(65–71)	71	(69–74)

### Pooled Age and Sex-Adjusted Stroke Death Rates (2011–2017)

**FIGURE 3:**
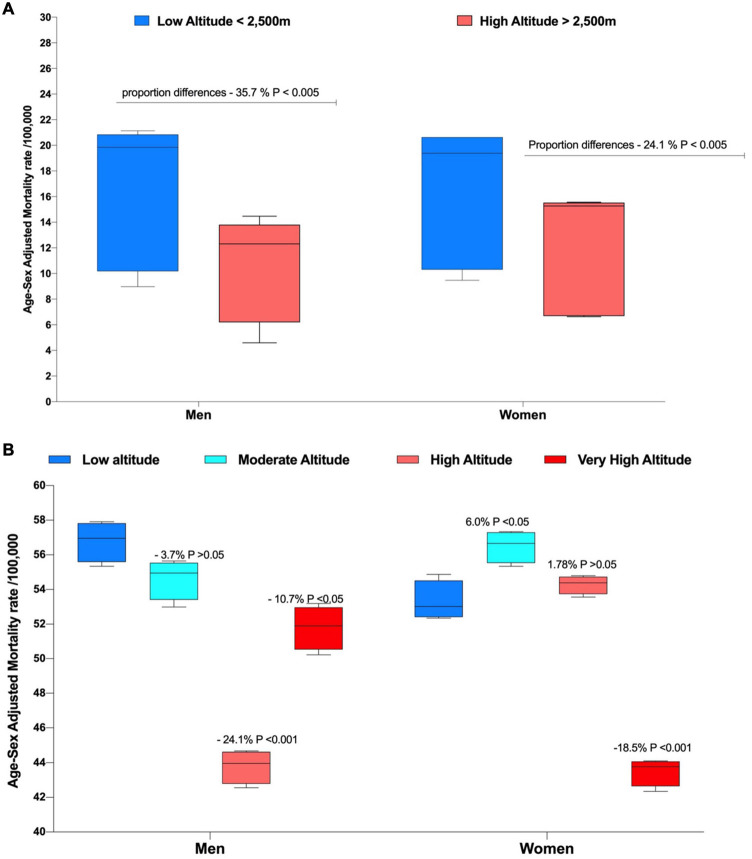
Overall age and sex-adjusted mortality rate by sex and elevation exposure. **(A)** Low altitude (<2,500 m) and High altitude (>2,500 m) Classification and **(B)** Low altitude (<1,500 m), Moderate altitude (1,500 − 2,500 m), High altitude (2,500 m − 3,500 m) and Very High altitude (3,500 m − 5,500 m).

In terms of mortality, when the age-sex adjusted, rates were applied to the low (<2,500 m) and high (>2,500 m) altitude population, the results demonstrate that the mortality rate is greater for the low altitude group in men [16.5/100,000 (CI 95% 11.5–21.4)] and women [16.2/100,000 (CI 95% 11.7–20.8)] versus [10.6/100,000 (CI 95% 6.9–14.3)] and women [12.3/100,000 (CI 95% 8.45–16.9)]. After computing the differences of proportions, men living at high altitude (>2,500 m) have 35.7% lower mortality rates than those living at lower altitudes (<2,500 m) and that this difference is greater in men than women (Figure 3A). When using the four-categories classification we found that men living at low altitude [56.7/100,000 (CI 95% 54.9–58.6)], moderate altitude [54.6/100,000 (CI 95% 52.7–56.4)], high altitude [43.7/100,000 (CI 95% 42.3–45.3)] and very high altitude [51.8/100,000 (CI 95% 49.7–53.8)] have higher stroke mortality than women at low altitude [53.3/100,000 (CI 95% 51.4–55.1)] and very high altitude [43.4/100,000 (CI 95% 42.2–44.7)]. In relation to the four altitude categories, we found that men residing at high and very high altitude have 24.1% and 10.7% lower mortality rates than their low altitude peers, and that these differences hold for both men and women (Figure 3B).

### Age and Sex-Adjusted Stroke Mortality and Stroke Admission Rates by Age Groups

**FIGURE 4:**
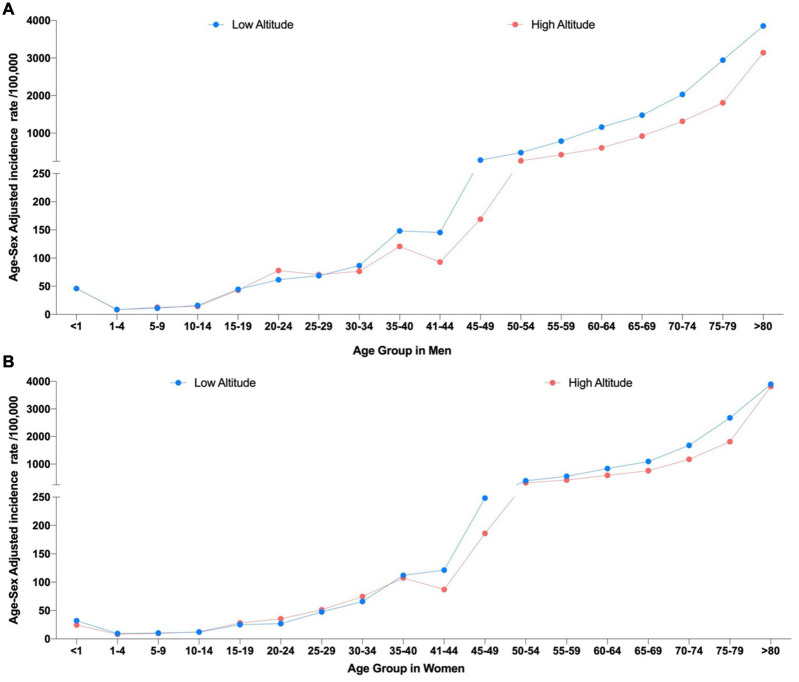
Age-sex adjusted incidence rates due to stroke by age group using the two elevation categories. **(A)** Incidence rates in men at low altitude (<2,500 m) and high altitude (>2,500 m) and **(B)** Incidence rates in women at Low altitude (<2,500 m) and High altitude.

Stroke hospital admission rates by age groups are significantly lower among younger population (<40 years of age) (Figure 4).

**FIGURE 5:**
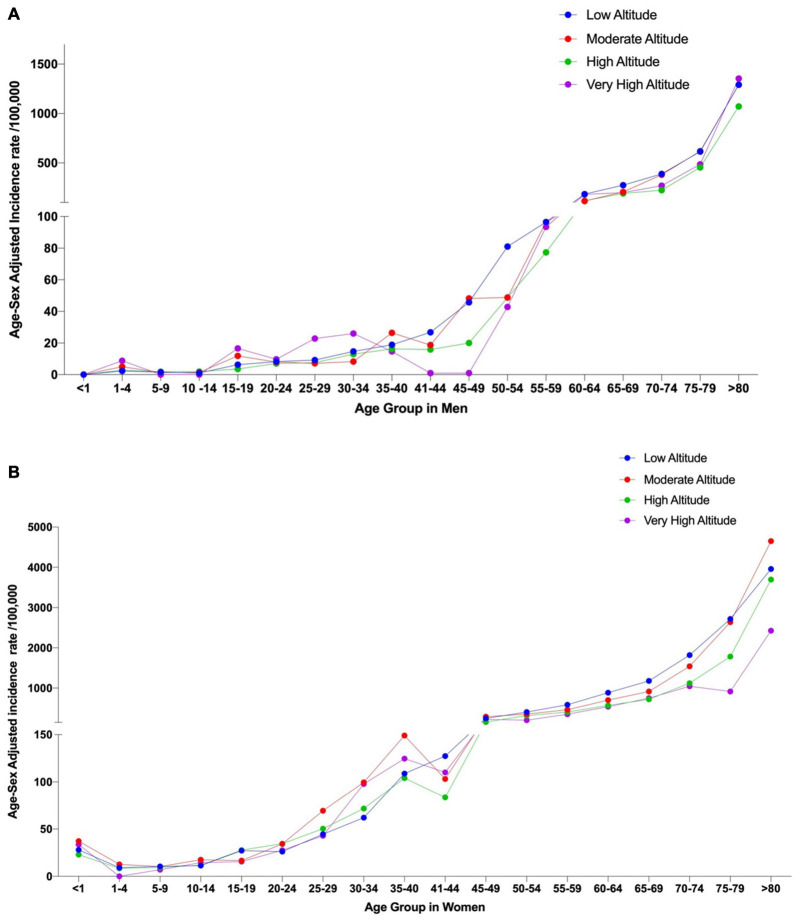
Age-sex adjusted incidence rates due to stroke by age group using the four elevation categories. **(A)** Incidence rates in men at Low Altitude (<1,500 m), Moderate Altitude (1,500 − 2,500 m), High altitude (2,500 m − 3,500 m) and Very High altitude (3,500 m − 5,500 m) and **(B)** Incidence rates in women at Low Altitude (<1,500 m), Moderate Altitude (1,500 m − 2,500 m), High altitude (2,500 m − 3,500 m) and Very High altitude (3,500 m − 5,500 m).

Differences among elevations groups did not yield statistically significant diffidence (*p* > 0.05) (Figure 5).

Age-sex adjusted rates at different elevations demonstrated that mortality and admission hospital rates per 100,000 people are greater for the low and moderate altitude groups in men and women when compared to the high and very high altitude groups (Table 2).

**TABLE 2:** Age-sex adjusted mortality and hospital admission stroke rates in Ecuador and the absolute differences in rates when compared to the reference elevation (Low altitude).

	Deaths			Hospital admission		
	(*n*)	Age-adjusted rates	Differences	(*n*)	Age-adjusted rates	Differences
**Men**						
Low altitude (<1,500 m)	11,241	57.9	Ref	25,003	305.4	Ref
Moderate altitude (1,500–2,500 m)	1,887	55.6	−2.3	4,412	255.9	−49.4
High altitude (2,500–3,500 m)	5,674	44.4	−13.5	9,921	196.8	−108.6
Very high altitude (3,500–5,500 m)	361	53.1	−4.8	233	170.0	−135.4
Total	19,163	N/A	−7	39,569	N/A	−98
Low altitude (<2,500 m)	13,344	56.3	Ref	29,652	291.6	Ref
High altitude (>2,500 m)	5,819	47.4	−8.9	9,917	204.3	−87.3
Total	19,163	N/A	−8.9	39,569	N/A	−87.3
**Women**						
Low altitude (<1,500 m)	9,770	53	Ref	20,371	251.6	Ref
Moderate altitude (1,500–2,500 m)	2,222	57.2	4.2	4,657	259.2	7.6
High altitude (2,500–3,500 m)	6,690	54.8	1.8	11,096	200.7	−50.9
Very high altitude (3,500–5,500 m)	356	44.1	−8.9	200	164.5	−87.1
Total	19,038	N/A	−1	36,324	N/A	−43.5
Low altitude (<2,500 m)	12,183	52.6	Ref	25,312	246.5	Ref
High altitude (>2,500 m)	6,855	56.9	4.3	11,012	208.5	−38.0
Total	19,038	N/A	4	36,324	N/A	−37.9

### Age-Specific and Sex-Specific Stroke-Related Hospital Admission and Mortality Risk in Ecuador

In the last 17 years of available data, we can observe that hospital admission is less likely to occur in the highlands for men OR: 0.69 CI 95% (0.68–0.71) and women OR: 0.83 CI 95% (0.83–0.86), while at 2,500 m of elevation and above, mortality risk was only reduced among men OR: 0.84 CI 95% (0.81–0.87) but no among women 1.08 CI 95% (1.05–1.12).

#### Stroke Mortality and Hospital Admission Relative Risk at Four Different Elevation Ranges

**FIGURE 6:**
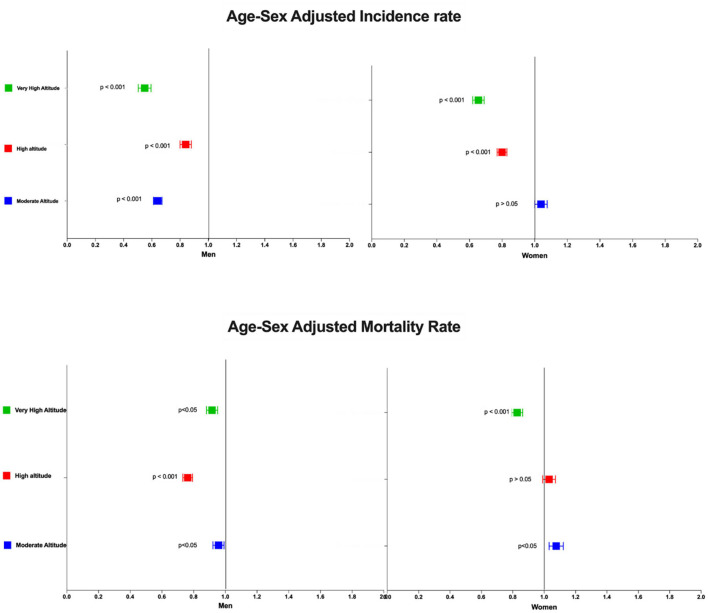
Cumulative risk for mortality and hospital admissions in men and women living at four different elevation in Ecuador from 2001 to 2017. Low altitude was used as a reference variable.

Populations from very high altitude are less likely to die due to stroke in both, men [OR: 0.91 (0.88–0.95)] and women [OR: 0.83 (0.79–0.86)]. Getting admitted to the hospital is also less likely to occur in the high altitude group [OR: 0.55 CI 95% (0.54–0.56)] when compared with the low altitude group OR: 0.65 CI 95% (0.64–0.66) (Figure 6). Using the International Society of Mountain Medicine classification, the probability of being admitted to the hospital was lower for the high [OR: 0.64 CI 95% (0.61–0.67)] and very high altitude group [OR: 0.55 CI 95% (0.50–0.59)] (Figure 6). While the probability of dying due to stroke was lower for men living at moderate [OR: 0.96 CI 95% (0.92–0.99)], high [OR: 0.76 CI 95% (0.73–0.79)] and very high altitude [OR: 0.91 CI 95% (0.88–0.95)] and lower for women living at very high altitude [OR: 0.83 CI 95% (0.79–0.86)] (Figure 6).

### Burden of Diseases Analysis

In terms of years of life lost prematurely (YLL), stroke predominantly caused mortality among older adults, especially men. From 2001, at least 109,759 years of life were lost prematurely due to stroke, 57,521 (52%) among men and 52,238 (47%) in women. Computing differences in rates yielded a lower burden of stroke at high and very high measured in YYL per 100,000 people (Table 3).

**TABLE 3:** Burden of stroke and years of life lost (YLL) prematurely due to stroke in Ecuador from 2001 to 2017.

Altitude	Population	Deaths	Death’s rate/1,000 pop.	YLL	YLL per 1,000 pop.
Low altitude (<1,500 m)	8,941,296	3,543	0.40 (0.38–0.41)	75,823	8.48 (8.42–8.54)
Moderate (1,500 − 2,500 m)	1,424,273	537	0.38 (0.35–0.41)	11,004	7.73 (7.58–7.87)
High altitude (2,500 m − 3,500 m)	4,360,711	1,249	0.29 (0.27–0.30)	21,698	4.98 (4.91–5.04)
Very high altitude (3,500 m − 5,500 m)	252,194	84	0.33 (0.27–0.41)	1,234	4.89 (4.62–5.17)
Total	14,978,474	5,413	0.36 (0.35–0.37)	109,759	7.33 (7.28–7.37)

*The most likely value at 95% confidence level (<95% less likely >95% less likely).*

### Access to Health Services at Different Elevations

The number of doctors, beds and hospital per population living at every single category of elevation is displayed in Table 4. In the four categories classification, the very high altitude range has 334 doctors per 100,000 people living at that elevation and 24 beds per 100,000 which is 86% less beds per capita that the high altitude group, 85% less than de moderate altitude group and 81% less than the low altitude group (Table 4).

**TABLE 4:** Number of hospital’s beds, number of doctors, and number of hospitals per 100,000 people living in Ecuador by different elevation range.

Altitude ranges	Hospital beds per 100,000	Doctors per 100,000	Hospitals per 100,000
Low altitude (<2,500 m)	130	452	3.0
Moderate altitude	162	518	2.5
High altitude (>2,500 m)	177	436	3.4
Very high altitude	24	334	2.6
Low	128	438	2.8
High altitude	173	435	3.2

In terms of doctors per capita, we found that at moderate elevation, the number of doctors per every 100,000 people is 518 per 100,000, having the low altitude group 12.7% less doctors per 100,000 people, followed by the high altitude group with 15.8% and the very high altitude group with 35.5% less doctors per 100,000 than the moderate altitude group (Table 4).

## Discussion

To our best knowledge, our study is the first to describe the burden of stroke at four different elevation ranges including low-moderate, high, and very high altitude. Ecuador is unique for this type of analysis since it has populations residing from sea level to very high altitude (4,300 m) (Figure 2). Understanding the country-wide distribution of stroke in Ecuador has already important implications in terms of evaluating the burden of this condition in a country where it has been poorly described before. Significant epidemiological differences in terms of stroke incidence and mortality were found when compared to the population settled at lower altitudes. Our results suggest that living at higher altitudes is associated with a reduction in the probability of developing ischemic stroke. The nationwide data from all the 221 elevations show that both men and women have fewer hospitalizations and lower incidence rates of stroke than their low-altitude controls. Stroke-related deaths are also lower within the high altitude group and the burden of stroke measured in years of life lost (YLL) due to premature mortality is also lower at higher altitudes.

Our results suggest that living at higher altitudes is associated with a risk reduction of developing stroke, evidenced by the significant lower rates of stroke admissions and mortality rates among the high altitude group, despite having similar access to health services. This results are similar to those reported in Switzerland in 2009, that study longitudinally compared stroke mortality at high altitude (Faeh et al., 2009). With increasing altitude, they found an almost constant decline in CHD and stroke mortality in Switzerland within a range of 259–1,960 m. These findings were more evident in men than in women, and the negative association between altitude and disease was stronger for CHD than for stroke (Faeh et al., 2009). Another epidemiological study recently published by Burtscher et al. (2021b), provided additional data supporting the statement that living at moderate altitude (1,000–2,000 m) elicits beneficial effects on all-cause mortality for both sexes, including diseases of the circulatory system in Austria. They found that cardiovascular diseases were main contributors to lower mortality rates at higher altitude within a 10-years period within this country (Burtscher et al., 2021b).

In terms of our findings, we observed a consistent dose-response relationship between high altitude and a reduced risk of developing stroke between 2,000 and 3,500 m of elevation. Beyond this point, other factors might be involved in a reduction of this hypothesized protective effect. For instance, it seems evident that living at certain elevation confers some risk reduction in terms of stroke incidence and stroke-related mortality (Faeh et al., 2009, 2016; Burtscher et al., 2021b).

Additionally, a well-performed comprehensive review, recently published by Burtscher et al. (2021a, b), emphasizes the relationship between hypoxia and brain aging, another important factor that might play a neuroprotective role (Burtscher et al., 2021a). For instance, they described cellular and physiological adaptations during intermittent hypoxia conditioning (IHC), rendering organisms more resistant to subsequent hypoxic or ischemic insults (Mayfield et al., 1994; Dudnik et al., 2018; Burtscher et al., 2021a). They also explored the facts around whether hypobaric or normobaric hypoxia could act as a neuroprotective factor, improving brain aging at high altitude. It seems that despite the importance of O_2_ in oxidative metabolism, aged organisms can profit from modestly hypoxic environments (Kim et al., 2011). They also described molecular pathways that might oppose aging, mainly driven by the Hypoxia-inducible factor 1 (HIF-1) that mediates the metabolic adaptation to hypoxia and ischemia, including the transition from oxidative to glycolytic metabolism as well as behaving as a positive modulator of aging (Leiser and Kaeberlein, 2010; Cerychova and Pavlinkova, 2018; Burtscher et al., 2021a).

Despite the vast amount of biomedical information related to high altitude hypoxia, ischemia and stroke, is still difficult to distinguish a relationship between biological protective factors (i.e., increased capillary vascularity and capillary perfusion per mm^3^ of brain tissue), better lifestyle habits or due to lower occurrence of well-known risk factors (i.e., smoking, obesity, hypertension) (Ortiz-Prado et al., 2010; Hill et al., 2011; Dunn et al., 2012; Hirschler et al., 2012; Voss et al., 2014; Steinback and Poulin, 2016; Beaudin et al., 2017; Liu et al., 2021).

For instance, brain angiogenesis is a common finding among acclimatized and adapted brain to high altitude (Ortiz-Prado et al., 2010; Dunn et al., 2012; Corante et al., 2020). This is the postulated most important protective factor when reducing the size of a stroke and when improving recovery after an ischemic episode at high altitude (Yang et al., 2010; Dunn et al., 2012; Hayakawa et al., 2017; Belayev et al., 2018). Nevertheless, above this point, high hematocrit levels due to a significant polycythemia causing thicker blood overcome this protective factor, reducing blood flow, and causing stasis as well as thrombogenesis (Tohgi et al., 1978; Meifang et al., 2000; Stavropoulos et al., 2017; Warny et al., 2019). Although no data from human studies are available, our results support the plausible relationship of a “protective window” that lays between 2,000 and 3,500 m of elevation, we suggest that anything below this point progressively loses the hypothesized protective effect that causes higher stroke incidence. On the other hand, anything above 3,500 m confronts angiogenesis and vascular remodeling with significant polycythemia, blood stasis and reduced blood flow, progressively triggering thrombogenesis.

It is well described that thrombogenesis is triggered by endothelial damage, blood stasis and increased coagulation (Gupta et al., 2019). When humans are exposed to altitudes greater than 3,500 m, the standard barometric pressure is 67 kPa (505 mmHg) and keeps getting low as we ascend (Ortiz-Prado et al., 2019). At this point, there is 66% of the oxygen available at sea level and the degree of hypoxia experienced relies on our adaptation (Frisancho, 1975; Beall, 2006). Nevertheless, and despite adaptation or acclimatation, either systemic or local hypoxia are inevitable at some point. Thrombosis and embolism at high altitude has been reported several times (Kotwal et al., 2007; Abeysekera et al., 2012; Parsehian et al., 2015; Singhal et al., 2016). The mechanisms related to this proposed prothrombotic state are numerous. Hypoxia is not only a consequences of vascular occlusion but also stimulates thrombogenesis (Tyagi et al., 2014). Polycythemia triggered by chronic high altitude exposure is an independent risk factor for thrombosis as well as for reducing and altering blood flow and cytokine-mediated inflammation in polycythemia (Brill et al., 2013; Zavanone et al., 2017; Jain et al., 2021; Jiang et al., 2021). At high altitude, other risk factors might also increase as elevation is gained. At high altitude, physical activity is reduced due to harsh conditions, reducing mobilization, and increasing hyperventilation (Sherpa and Shrestha, 2020; Uno et al., 2020). Dehydration aggravating blood stasis, hyperventilation aggravating dehydration and a proposed increased platelet adhesiveness might counteract some of the protective effects related to a reduced stroke incidence found at moderate or high altitude (Sharma et al., 1980; Tyagi et al., 2014). Although we do not know the elevation threshold at which high altitude becomes a stroke risk factor, we suggest that above 3,500 m, especially in unacclimatized individuals the protective effect starts to fade.

In relation to high altitude lifestyle differences, habits, and endogenous preconditioning, gathering data is a complex task. Different populations have different eating habits, different lifestyles and they usually subsist in a different way than their low-land counterpart (Westerterp, 2001; Lundby et al., 2006; Li and Zhao, 2015; Brutsaert, 2016). The data about risk factors available in Ecuador suggest that people living in provinces from the highlands consume more alcohol (17.1% versus 9.1%) and smoke more (6.5% versus 2.5%) than the people living at lower altitudes (Freire et al., 2015). These behaviors might contradict the paradoxically lower mortality of stroke among highlanders (INEC, 2015). People from the coast seem to have higher consumption of carbohydrates (36% versus 30%) and have inferior access to health services (24 versus 32 health centers per every 100,000 people) than the persons in the highlands (INEC, 2018). Although these data on dietary variability could be extrapolated to the population living at high altitude in Ecuador, it is well-known that people visiting high altitude locations have a significant loss in appetite as well as an accelerate metabolism that might speedup weight loss (Karl et al., 2018; Rausch et al., 2018).

In terms of diabetes, there is no published information about the differences for high and low altitude population in Ecuador, nevertheless, the crude mortality rate according to the INEC database. We found only one study that showed that diabetes in the coastal provinces is more prevalent (388/100,000) than in the highlands (236/100,000), situation that is similar to other studies (Santos et al., 2001; Lopez-Pascual et al., 2018). The available literature suggests that short-term exposure to high altitude leads to transient hyperglycemia, primarily triggered by activation of the sympathetic system, while long-term exposure results in lower plasma glucose concentrations, mediated by better insulin sensitivity and increased clearance of peripheral glucose (Koufakis et al., 2019). An inverse relationship between altitude, diabetes, and obesity has been well documented. Woolcott in their study showed that living at high altitude (1,500–3,500 m) is associated with a lower likelihood of having diabetes than living between 0 and 499 m in the same way those living at high altitude were 25% less likely to be obese (Woolcott et al., 2014). Several hypothesis have been raised about weight loss at high altitude, including those briefly discussed above (increased resting metabolic rate), as well as the possible role of endocrine molecules such as neuropeptide Y (NPY), ghrelin, galanin, cholecystokinin (CCK), and interleukin-6 (IL-6) stimulating weight loss (Duraisamy et al., 2015). In addition, the negative energy balance in hypoxia seems to be largely due to a reduction in energy intake due to lack of appetite and probably due to a reduced carbohydrate and lipids intake (Díaz-Gutiérrez et al., 2016).

Other risk factors such as hypertension and sedentarism have been showed to be less prevalent among high altitude dwellers according to some of the available published data (INEC, 2009; Ortiz-Prado and Dunn, 2011; Narvaez-Guerra et al., 2018). Although a cause-effect relationship between high altitude and stroke cannot be established with ecological studies, our results suggest that milder chronic hypoxia could play a protective role for the development of stroke, and more severe long-term hypoxia (>3,500 m) might be linked to a higher burden of cerebrovascular diseases (Pilz et al., 2008; Faeh et al., 2009; Kent et al., 2013; Lopez et al., 2018).

The observed results show an apparent protective effect of residing at high altitude in relation to stroke. In that sense, there are some biases that we cannot control due to limitations of the data itself. For instance, the healthy user effect bias is one that we could not controlled. There is no mechanism to discern if one patient represents two cases receiving medical attention in two different places at two different times. We have already discussed about the differences in terms of exercise, diet, or habits between the two populations and although we have information available, not all variables can be controlled with an observational study design such as this. Despite these limitations, we believe that the impact of these biases in a setting such as ours should not have a major effect. Similar results have been obtained in well- controlled studies, suggesting important insights into the role that chronic hypoxia and stroke-associated survival at high altitude.

## Limitations

The main limitation of this analysis is that some residual risk factors such as educational attainment, socioeconomic status, BMI, BP, smoking, diabetes, and polycythemia were not controlled since data is not available. Another limitation is the lack of information needed to run a proportional hazards model to estimate survival rates. The data available in the country do not refer to the time that passed before any of the events occurred (in this case stroke related deaths) and we only have information on how many of those events were recorded on a giving year.

Observational epidemiological studies, especially ecological ones, cannot conclude a cause-effect relationship, however, they are very valuable for identifying associations and relationships for further investigations.

## Conclusion

This is the first epidemiological study that attempt to explore the association between stroke and the burden of cerebrovascular disease at different elevations, ranging from 0 to 4,300 m above sea level. Our findings suggest that living at higher elevations might offer a reduction of the risk of dying due to stroke as well as a reduction in the probability of being admitted to the hospital. Nevertheless, this finding has the strongest epidemiological repercussions between 2,000 and 3,500 m and beyond this point, the hypothesized protective effect starts to fade. It could be hypothesized that high altitude residents, probably due to their increased cerebral microvasculature have better perfusion, thus protecting the brain in some degree against the hypoxic insult. Although the results of this ecological study support a previously proposed theory, more investigation is needed to understand the complex relationship between hypobaric hypoxia, time of exposure and stroke.

## Data Availability Statement

The original contributions presented in the study are publicly available. This data can be found here https://github.com/covid19ec/StrokeData.

## Ethics Statement

This secondary data analysis of publicly available, anonymized data received Ethical Approval from the University of Southampton with the Faculty of Medicine Ethics Committee ERGO 51422.R3 number.

## Author Contributions

EO-P contributed toward the conception and design of the whole project, obtained full access to the data from the National Institutes of Statistic in Ecuador, was primarily accountable for all aspects of work, and ensuring integrity and accuracy of the research as well as of the drafting of the manuscript. SC, JV, and AB-G contributed toward data acquisition and revision of the available literature. KS-R, LG-B, and MC-A contributed to the statistical analysis, internal validity of the study, and initial drafting of the manuscript. AH-T undertook the burden of disease analysis and economic impact of stroke at different altitudes in Ecuador. PE, AB, GV, and PR critically reviewed and edited the manuscript, and providing input toward the reporting of the data and its interpretation. All authors contributed to the article and approved the submitted version.

## Conflict of Interest

The authors declare that the research was conducted in the absence of any commercial or financial relationships that could be construed as a potential conflict of interest.

## Publisher’s Note

All claims expressed in this article are solely those of the authors and do not necessarily represent those of their affiliated organizations, or those of the publisher, the editors and the reviewers. Any product that may be evaluated in this article, or claim that may be made by its manufacturer, is not guaranteed or endorsed by the publisher.
